# Two novel mutations identified in familial cases with Donohue syndrome

**DOI:** 10.1002/mgg3.43

**Published:** 2013-11-14

**Authors:** Tzipora C Falik Zaccai, Limor Kalfon, Aharon Klar, Mordechai Ben Elisha, Haggit Hurvitz, Galina Weingarten, Emelia Chechik, Vered Fleisher Sheffer, Raid Haj Yahya, Gal Meidan, Eva Gross-Kieselstein, Dvora Bauman, Sylvia Hershkovitz, Yuval Yaron, Avi Orr-Urtreger, Efrat Wertheimer

**Affiliations:** 1 Institute of Human Genetics, Western Galilee Medical CenterNaharia, Israel; 2 Faculty of Medicine in Galilee, Bar-Ilan UniversitySafed, Israel; 3 Department of Pediatrics, Bikur Cholim General Hospital, affiliated with the Hebrew University-Hadassah Medical SchoolJerusalem, Israel; 4 Department of Neonatology, Western Galilee Medical CenterNaharia, Israel; 5 Department of Pathology, Sackler School of Medicine, Tel Aviv UniversityTel Aviv, Israel; 6 Sherutei Briut ClalitWestern Galilee District, Israel; 7 Department of Obstetrics and Gynecology, Bikur Cholim General HospitalJerusalem, Israel; 8 Genetics Institute, Tel Aviv Sourasky Medical Center, Sackler School of Medicine, Tel Aviv UniversityTel Aviv, Israel

**Keywords:** Cardiomyopathy, Donohue syndrome, genotype–phenotype, insulin receptor.

## Abstract

Donohue syndrome (DS) is a rare and lethal autosomal recessive disease caused by mutations in the insulin receptor (*INSR*) gene, manifesting marked insulin resistance, severe growth retardation, hypertrichosis, and characteristic dysmorphic features. We report the clinical, molecular, and biochemical characterization of three new patients with DS, and address genotype–phenotype issues playing a role in the pathophysiology of DS. A female infant born to first-degree cousins Muslim Arab parents and two brothers born to first-degree cousins Druze parents presented classical features of DS with hypertrophic cardiomyopathy and died in infancy. Each patient was found homozygous for one missense mutation within the extracellular domain of the *INSR* gene. Western blot analysis identified the proreceptor of INSR, but not its mature subunits alpha and beta. Of 95 healthy Muslims, no heterozygous was found and of 52 healthy Druze from the same village, one was heterozygous. This study presents two novel familial mutations in the alpha subunit of the *INSR* which appear to impair post-translational processing of the INSR, resulting loss of its function. Both mutations cause DS with hypertrophic cardiomyopathy and early death. Identification of the causative mutation enables prevention of this devastating disease.

## Introduction

Single-gene defects are responsible for hyperglycemia in only a minority of individuals. Mutations affecting both insulin production and insulin sensitivity have been identified. Maturity onset diabetes in the young is a monogenic type of diabetes in which a mutation in an autosomal dominant gene disrupts insulin production (Winckler et al. 2007). Donohue syndrome (DS, OMIM#246200), also known as Leprechaunism, Rabson–Mendenhall syndrome, and type A insulin resistance are autosomal recessive (AR) disorders caused by biallelic mutations in the gene encoding the insulin receptor (*INSR*, OMIM#147670). These syndromes, sharing phenotype and genotype heterogeneity, are distinguished from one another based on the severity of symptoms, age of onset, and age of death (Porter and Barrett 2005). DS is considered the most severe syndrome of the group, and is usually lethal within 2 years of life (Musso et al. 2004; Semple et al. 2011; Grasso et al. 2013).

Donohue syndrome is characterized by markedly delayed linear growth and failure to thrive (FTT), loss of glucose homeostasis, hyperinsulinemia, thick skin with lack of subcutaneous fat, acanthosis nigricans (AN), distended abdomen, enlarged genitalia in the male and cystic ovaries in the female, and dysmorphic facial features: elfin faces with prominent eyes, thick lips, upturned nostrils, and low-set posterior rotated ears (Geffner et al. 1987; al-Gazali et al. 1993).

A wide spectrum of disorders caused by diverse monogenic etiologies resembles DS. Specifically, Berardinelli–Seip congenital lipodystrophy is a condition associating insulin resistance, absence of subcutaneous fat, AN, and muscular hypertrophy caused by mutation in either *AGPAT2* (OMIM#603100) or *BSCL2* (OMIM# 606158) (Friguls et al. 2009; Miranda et al. 2009), AN associating with severe skeletal dysplasias due to activating mutations in *FGFR3* (OMIM#134934) (Alatzoglou et al. 2009) are just a few examples.

The association between diabetes and cardiovascular disease is well recognized (Kannel and McGee 1979). Furthermore, evidence for insulin resistance has been shown to associate with cardiovascular disease, and specifically, with hypertrophic cardiomyopathy (HCM), also in individuals without diabetes (Murakami et al. 2004; Bonora et al. 2007; Verhagen et al. 2011). Recently, a large meta-analysis substantiated the association between metabolic syndrome and cardiovascular disease (Mottillo et al. 2010). Insulin resistance is a central component of both metabolic syndrome and DS. Several reports (Baykan et al. 2008; Nobile et al. 2012; Hovnik et al. 2013) argue that the underlying mechanisms for cardiomyopathy in DS and metabolic syndrome involve excess insulin activation of Insulin-like growth factor 1 (IGF1) receptors [6].

In this study we characterized two novel missense mutations in the *INSR* causing DS with HCM.

## Material and Methods

### Patients

The patients were three newborns from two unrelated families who were hospitalized in neonatal intensive care units: a female born to first-degree cousins Muslim Arab parents (named patient ISR1) and two brothers with first-degree cousins Druze parents (named patients ISR2 and ISR3). The IRB of Nahariya Medical Center and the Israeli Ministry of Health approved the study.

### Clinical examination

Family history was taken and physical examinations conducted. Imaging studies of the brain, abdomen, and heart were performed. Biochemical workup included renal and liver function tests, glucose, insulin, C-peptide, and glucagon levels. Blood was drawn for molecular studies, and fibroblast cells cultures were established from skin biopsies of patients ISR1 and ISR2 as previously described (Falik-Zaccai et al. 2008b).

### Mutation analysis in the insulin receptor gene

#### For patient ISR 1

Studies were carried to locate the *INSR* underlying mutation by denaturing high-performance liquid chromatography (dHPLC) of each of the 22 INSR exons, including exon-intron boundaries. Exons demonstrating abnormal pattern, compared with control, in the dHPLC screening were further sequenced for determining possible DNA polymorphism. Results were confirmed using *Allele-specific oligonucleotide* (*ASO*) using radiolabelled oligoprobes: 5′-TCAGCTTCTGCCAGGACC-3′ (wild type) and 5′-TCAGCTTCTACCAGGACC-3′ (mutant). Population screening of 95 Arab Muslims was carried out using Sanger sequencing of a 347 bp amplicon containing the p.C286Y mutation (Macrogen Inc, Amsterdam, Netherlands).

#### For patients 2 and 3

The 22 exons of the *INSR* (NM_000208.2) were Sanger sequenced and analyzed using the ABI PRISM 3130 Genetic Analyzer (Applied Biosystems, Warrington, U.K.) according to the manufacturer's instructions.

For the Druze patients, sequencing results were confirmed and healthy controls were examined by chain reaction (PCR) amplification of exon 2 using the primers:

INSR_ex2 (1) F: 5′- GAT GAA AAC ACA GGG CCC AG- 3′

INSR_ex2(1) R: 5′- CTC CAC GGA ATC CAG GAT AC -3′

The reaction was followed by enzymatic digestion using the restriction enzyme TaqI (New England Biolabs, Ipswich, MA).

### Immunoprecipitation and immunoblotting analysis

Protein lysate was prepared according to standard protocols and studies were carried out as detailed elsewhere (Wertheimer et al. 1993). Antibodies used for immunoblotting and immunoprecipitation procedures included monoclonal antibody recognizing phosphotyrosine residues (Upstate Biotechnology, Inc., Lake Placid, NY) and rabbit polyclonal antibodies against the insulin receptor beta subunit (Santa Cruz Biotechnology, Inc., Santa Cruz, CA).

### Prenatal diagnosis

Prenatal diagnosis (PND) was performed for the Arab Muslim family. After comprehensive genetic counseling, chorionic villous sampling (CVS) was performed. DNA was extracted from the villi and exon 3 of the *INSR* gene sequenced.

## Results

### Patient characteristics

All three patients presented with the classical elfin features characteristic of DS: coarse face, bulging eyes, and thick lips, absence of subcutaneous fat, hirsutism, and nipple hypertrophy. Laboratory tests revealed direct hyperbilirubinemia, and elevated Gamma Glutamyltransferase. Other liver and kidney function tests were normal. All patients suffered from intrauterine growth restriction (IUGR), FTT, and HCM.

**Figure 1:**
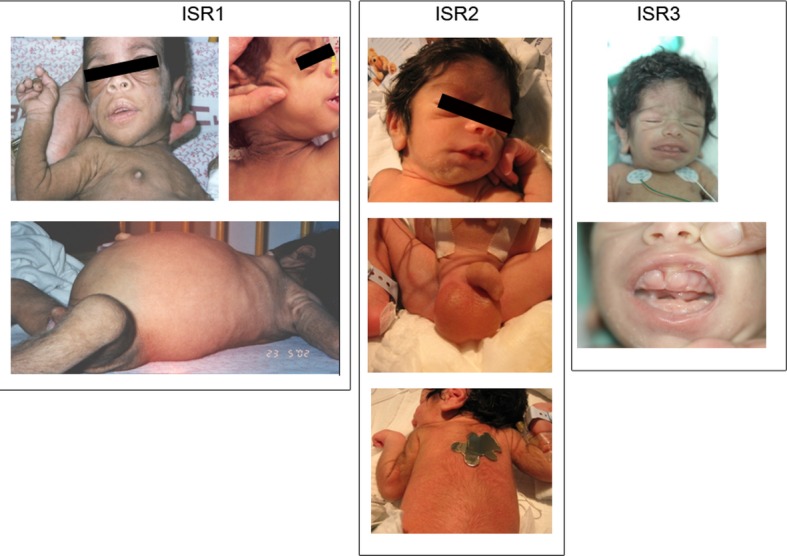
Clinical features of patients. See description in text.

The diagnosis of DS was established based on the above clinical characteristics, and determination of the *INSR* mutation. The particular features and disease course, and the biochemical tests of the patients, are depicted in Figure 1 and summarized in Table 1, respectively.

**Table 1:** Clinical and laboratory characteristics of the three patients.

Patient number and sex (M/F)	Birth weight and gestational age	Head circumference	Insulin levels *N* = 5–25	C-peptide levels *N* = 298–1324	Age at death
ISR1 – F	1700 g (−2.2 SD); 36 weeks	32.5 cm (−2 SD) at 14 days	3560 IU/mL (age: 1 month)	15900 pmol/L (age: 1 month)	18 months
ISR2 – M	1540 g (−2 SD); 34 weeks	31 cm (−3 SD) at birth	4500 IU/mL (at birth)	Not available	40 days
ISR3 – M	1770 g (−2.5 SD); 38 weeks	32.6 cm (−2 SD) at birth	2761, 4300, 21525 IU/mL (at birth)	24825 pmol/L	12 months

N, normal.

#### Family 1

Patient ISR1, the second child of first-degree cousins Muslim Arab parents, presented at age 14 days with abdominal distention and restlessness. Her weight was 1.900 g (−2.2 SD). She presented with classical physical signs of DS including hypertrophy of the labia majora and clitoromegaly. Ultrasound of the abdomen showed ovaries with bilateral multicystic masses (10 × 10 × 20 mm; 7 × 13 × 15 mm) and enlarged kidneys.

The clinical course showed no weight gain, episodes of hypoglycemia, AN, distended abdomen, and rectal prolapse. Echocardiography revealed hypertrophy of the left ventricle. Abdominal ultrasound demonstrated enlarged kidneys with medullary calcinosis and further enlargement of the ovaries: L – 50 × 70 × 75 mm, and R – 20 × 40 × 45 mm. The following months were typified by recurrent infections including urosepsis, bilateral otitis media, and pneumonia, and failure to reach neurological milestones. At age 18 months the patient died due to aspiration pneumonia.

#### Family 2

Patient ISR2, the third child of first-degree cousins Druze parents, was born at 34 weeks gestation. He presented with IUGR, dysmorphic features, and hirsutism (Table 1), hepatosplenomegaly, and hypotonicity. Ultrasound examination during pregnancy revealed polyhydramnion and enlarged kidneys, bladder, and stomach. Ultrasound imaging of the abdomen revealed hepatosplenomegaly and enlarged kidneys.

The clinical course showed FTT, and episodes of alternating hypoglycemia and hyperglycemia. At age 3 days a 2–3/6 systolic heart murmur was heard. Echocardiogram revealed severe HCM associated especially with hypertrophy of the left ventricle and septum. DS was diagnosed based on the clinical characteristics, and the identified causative mutation in *INSR*. At 2 weeks Klebsiella sepsis was diagnosed and treated. At age 40 days the infant died due to respiratory failure and cardiac arrest.

**Figure 2:**
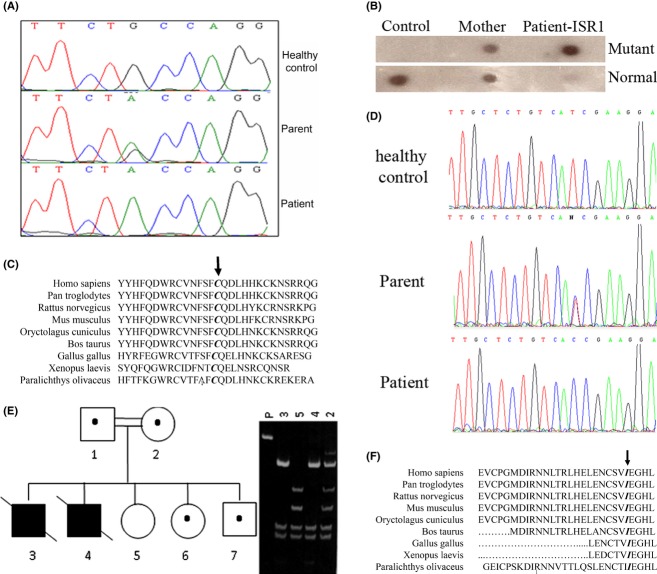
Mutation analysis. Genomic DNA sequence analysis revealed a dHPLC abnormal pattern of exon 3 of patient ISR1; the novel c.858G>A mutation was detected (A). The mutation was further confirmed by allele-specific oligonucleotide hybridization (B). High conservation of p.C286 throughout the phylogenetic tree (C). 3 genomic DNA sequence analysis of the 22 exons of *INSR* gene revealed a homozygous T > C transition at nucleotide 167 in exon 2 (D). The family pedigree was drawn according to restriction digestion with TaqI enzyme (E). Fragmented polymerase chain reaction (PCR) products were visualized by ethidium bromide-stained acrylamide gel. P, PCR product (E). The amino acid residues Cysteine at position 286 (C) and Isoleucine at position 56 (F) in the *INSR* are shown to be conserved throughout the phylogenetic tree. Amino acid conservation was analyzed by NCBI Basic Local Alignment Search Tool, using protein blast.

Patient ISR3, the brother of patient 2 (Fig. 2E), was born after an uneventful 38-week pregnancy, presenting symmetric IUGR. Ultrasound examination during pregnancy revealed an enlarged heart. He resembled his deceased brother both clinically and biochemically (Table 1). Echocardiography revealed hypertrophy of the left ventricle and mild pulmonic stenosis.

At age 3 months, the patient suffered from FTT (weight 3.1 kg), AN, distended abdomen, rectal prolapse, and a right inguinal hernia. Hypothyroidism was diagnosed at the age of 2 months and 50 *μ*g of eltroxin were administered daily. At age 7 months FTT was severe, and bilateral inguinal hernias hypotonia and severe cardiac hypertrophy were present. Liver enzymes were elevated. Clotting functions were abnormal, with factor 7 deficiency. MRI of the brain failed to demonstrate the neuronal pituitary gland. The stalk and the anterior part of the pituitary were normal. Maturation of white matter was described to be slow. At age 10 months urosepsis was diagnosed, and ampicillin and gentamicin were administered. Body weight was 4125 g (−6 SD) and head circumference 42 cm (−3 SD). At age 11 months right upper lobe pneumonia was detected. At age 12 months, the patient died following an episode of atrial fibrillation.

### Mutation analysis

Patient ISR1 was found to be homozygous for a novel missense mutation c.858G>A, substituting cysteine to tyrosine at position 286 (p.C286Y) in exon 3 of the *INSR* gene within the extracellular alpha subunit (Fig. 2A). This was confirmed by allele-specific oligonucleotide (Fig. 2B). p.C286 is found to be highly conserved throughout the phylogenetic tree, suggesting a strong functional value for this amino acid position (Fig. 2C).

Patient ISR3 was found to be homozygous for a novel missense mutation, T > C transition at nucleotide 167 in exon 2 (c.167T>C) of the *INSR* (Fig. 2D), resulting in isoleucine substitution to threonine at position 56 (p.I56T) within the extracellular alpha subunit. p.I56 is also found to be conserved throughout the phylogenetic tree, suggesting a strong functional value for this amino acid as well (Fig. 2F). Restriction enzyme analysis showed the mutation to be in full segregation in the patient's family. Patient ISR2 was found to be homozygous for the same mutation. Parents are heterozygous carriers, a healthy brother and sister are heterozygous carriers, and another healthy sister did not carry the mutation (Fig. 2E).

### Prenatal diagnosis

Prenatal diagnosis was performed for family 1 three times via CVS using sequencing analysis. One fetus was found to be homozygous for the causative mutation and the pregnancy was terminated. The two other fetuses were found to be heterozygous and two healthy babies were born as predicted.

### Population screening

Screening of 95 healthy Muslims revealed no heterozygous carriers for the c.858G>A mutation. Of 52 healthy individuals from the Druze patient's village, one carrier for the novel mutation c.167T>C was found.

### In vitro characterization of the insulin receptor

**Figure 3:**
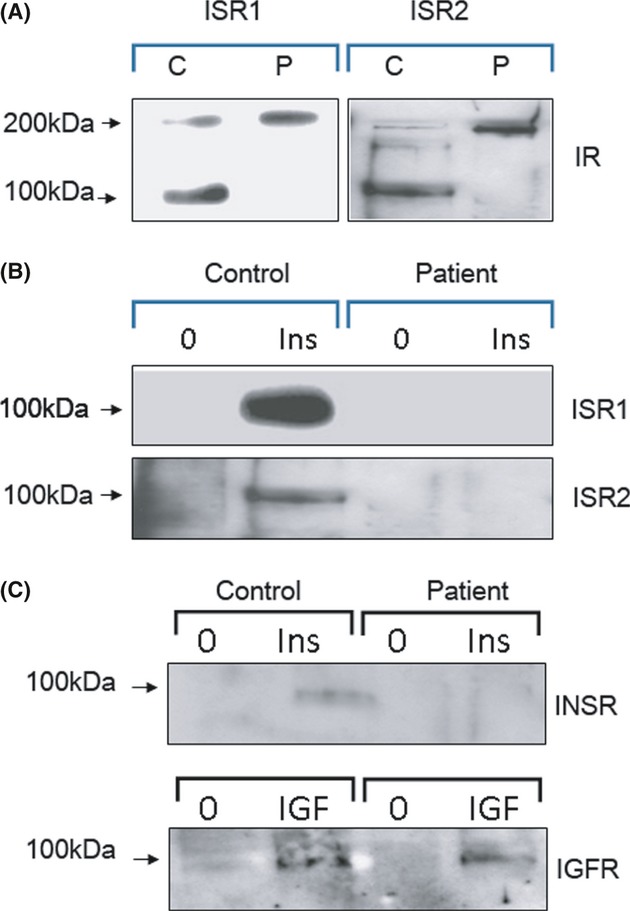
INSR determination and functionality. The proreceptor but not the mature subunits in the ISR1 and ISR2 fibroblasts (labeled P) is detected, compared with both forms identified in the healthy control (labeled C) (A). Fibroblasts were treated with 1 μmol/L of insulin (B) or IGF1(C), immunoprecipitated with an antibody against phosphorylated tyrosine, and immunoblotted with an antibody against INSR or IGF receptor, respectively.

To study the effect of the detected genetic alterations on INSR function, we established fibroblast cultures from skin biopsies taken from patients 1 and 3. Figure 3 shows absence of tyrosine phosphorylation at endogenous state (Fig. 3A), and following induction of INSR precipitation by insulin (Fig. 3B), indicating a lack of functionality of the receptor in the two DS fibroblasts compared to normal fibroblast. In comparison, it appears that the expression and phosphorylation of the closely related IGF-1 receptor in response to IGF-1 was normal (Fig. 3C).

To identify the reason for lack of INSR phosphorylation, we investigated INSR intracellular processing. Western blot analysis of INSR, revealed the proreceptor at position 210 kDa, but not the mature alpha and beta subunits (∼130 and 95–100 kDa, respectively), providing strong evidence that the two mutated amino acids disrupted the normal processing of INSR.

## Discussion

We have ascertained three new patients with DS based on their clinical appearance and the identification of two familial causative novel missense mutations in *INSR*. The phenotype of all three was similar including HCM, and survival did not exceed 18 months.

The INSR is transcribed as a single glycosylated precursor, which after transport from the endoplasmic reticulum to the Golgi apparatus is further glycosylated and then cleaved into extracellular alpha domains comprising ligand-binding activity and beta subunits, encompassing intracellular tyrosine kinase activity. These subunits are subsequently transported to the plasma membrane as a α2β2 heterotetramer (Seino et al. 1989).

The two novel missense mutations described herein are located in the extracellular domain of INSR, and led to impair processing of the receptor. According to the tertiary structure of the ectodomain of INSR, the mutation presenting in patients ISR2 and ISR3 (p.I56T) is located within the L1 domain, and the mutation presenting in patient ISR1 (p.C286Y) in the cysteine-rich domain (Desbois-Mouthon et al. 1997; McKern et al. 2006). The severe biochemical and clinical consequences of this latter mutation highlights the crucial role of the cysteine residues in the INSR ligand-binding domain, not only in ligand binding, but also in stabilizing the three-dimensional IR structure affecting intracellular INSR processing.

Phenotypic heterogeneity has been demonstrated in a number of documentations of DS (Semple et al. 2011). For example; One patient who carried a homozygous deletion of the entire *INSR* gene, and thus absolute lack of insulin receptor activity, survived for 3.5 years before dying from postoperative complications (Wertheimer et al. 1993), contrasting with another infant with almost no insulin receptor activity consequent to a nonsense mutation at position 121, who failed to thrive, and died at 16 weeks of age (Krook et al. 1993). These two patients present a most striking discrepancy between genotype and phenotype in DS. Both carried what seemed to be complete inactivation of the INSR protein; however, one survived longer than all expectations, whereas the other died after a very short period. Maassen et al. found that the degree of insulin binding among five patients with defects in the INSR did not correspond to the severity of the clinical phenotype (Maassen et al. 2003). These data suggest that the absence of the INSR protein is not identical to absence of the gene itself, and that activity of transcriptional factors may also have an important effect.

Among the 100 missense mutations reported to date (Stenson et al. 2009), phenotypic variability has been shown to occur. Both novel mutations described here lie in the extracellular region and contain the insulin-binding region and a cysteine-rich domain, presenting severe type of DS. In contrast, Ahmad et al. (2013) reported a c.659C>T substitution in exon 3, which is in the same domain of the INSR, presenting a mild phenotype of AN with normal fasting and postprandial blood glucose levels. The same mutation was reported by Carrera et al. (1993) to cause Rabson–Mendenhall syndrome.

The Phenotypic variability caused by single missense mutation has been suggested to be due to the coexistence of an additional sequence variation in a modifier gene. Candidate genes might be *BSCL2*, *AGPAT2*, *CAV1* (OMIM#601047), and *PTRF* (OMIM#603198), known to cause primarily generalized lipodystrophy with similar clinical manifestations of insulin resistance and hyperinsulinism, AN, and more (Rahman et al. 2013). Moreover, the phenotypic variability might result from various degrees of inactivation of *INSR* transcription, leading to diverse activation of compensatory pathways, as has been shown in many cases of protein inactivation in transgenic animals. If such a scenario exists in DS, the most likely compensatory pathway might be through the closely related IGF-1 receptor.

DS appears to result from either homozygous or compound heterozygous mutations. Approximately 130 mutations have been reported to be causative of DS so far (Stenson et al. 2009). As a classic AR trait homozygous or compound heterozygous mutations are expected to cause the phenotype while heterozygous individuals are healthy carriers and free of symptoms. This is the case in the two families reported here. However, there are reports of individuals heterozygous for other mutations in *INSR* who were symptomatic with hyperandrogenism, AN, hyperinsulinemia, and insulin resistance, as well as Rabson–Mendenhall and type A syndrome (Takahashi et al. 2010; Kadowaki et al. 1990; Wertheimer et al. 1994), suggesting again the possibility of a modifier gene involved in the phenotype.

In this study, all three patients presented cardiomyopathy with hypertrophy of the left ventricle and septum. The families did not consent to a histopathology analysis. HCM was previously documented as a manifestation of DS (Baykan et al. 2008); it has also been associated with less severe states of elevated insulin levels, such as in infants of mothers with diabetes (Russell et al. 2008). Furthermore, insulin resistance has been associated with left ventricular diastolic dysfunction (LVDD) in adults without diabetes (Dinh et al. 2010). Accumulating evidence suggests that reactive oxygen species (ROS) may have a role in insulin-resistant cardiomyopathy (Mellor et al. 2010) and that ROS may explain some of the pathophysiology of DS (Park et al. 2010). It is yet unclear if cardiomyopathy develops due to insulin resistance or to hyperinsulinemia. Investigation of congenital syndromes of impaired function in the insulin receptor may elucidate effects of insulin that are not known to be related to obesity or diabetes. We present here an example of HCM associated with mutations within the extracellular domain of the *INSR* gene. We do not know the degree to which the cardiomyopathy presenting in all three patients resulted in their early death. The congruence of insulin resistance and cardiomyopathy in the two novel mutations described here further supports a common genetic basis for insulin resistance and cardiovascular disease.

Parents of the three patients presented here are first-degree cousins who reside in villages with high rates of consanguineous marriages. Prevalence rates of rare AR diseases are high among the Druze and Arab Muslim populations (Falik-Zaccai et al. 2008a,b2008b, 2010). Each patient might be the tip of an iceberg indicating the presence of high-risk population for a devastating disease. Therefore, following identification of a new mutation in an isolated village we perform small-scale screening to identify high-risk populations for severe AR rare diseases (Falik-Zaccai et al. 2008a,b2008b, 2010). The fact that DS does not occur at elevated frequency in the Muslim and Druze population studied here suggests the identification of “private, familial” mutations.

High dosages of insulin (Casati et al. 2010) and recombinant human insulin-like growth factor-1 (rhIGF-1) (Kitamei et al. 2005) have been used to treat DS; the latter was considered to have played a role in the development of diabetes retinopathy (Kitamei et al. 2005). When the clinical course is fatal and treatment is not effective, as with our patients, then identification of the causal mutation and PND is of particularly importance. Characterization of the causative mutations enabled accurate genetic counseling and PND for the DS described.

Elucidation of correlations between genotypes and phenotypes of congenital syndromes of impaired function of the INSR may contribute to the understanding of the process of insulin action in both healthy and pathological states.

## Conflict of Interest

None declared.
